# Reduction of Emphysema Severity by Human Umbilical Cord-Derived Mesenchymal Stem Cells in Mice

**DOI:** 10.3390/ijms23168906

**Published:** 2022-08-10

**Authors:** Vincent Laiman, Yueh-Lun Lee, Yu-Wei Hou, Yu-Ting Fang, You-Yin Chen, Yu-Chun Lo, Didik Setyo Heriyanto, Shu-Chi Lan, Chia-Ling Chen, Xiao-Yue Chen, Kang-Yun Lee, Jer-Hwa Chang, Hsiao-Chi Chuang

**Affiliations:** 1 International Ph.D. Program in Medicine, College of Medicine, Taipei Medical University, Taipei 110, Taiwan; 2 Department of Anatomical Pathology, Faculty of Medicine, Public Health and Nursing, Universitas Gadjah Mada—Dr. Sardjito Hospital, Yogyakarta 55281, Indonesia; 3 Department of Microbiology and Immunology, School of Medicine, College of Medicine, Taipei Medical University, Taipei 110, Taiwan; 4 School of Respiratory Therapy, College of Medicine, Taipei Medical University, Taipei 110, Taiwan; 5 Department of Biomedical Engineering, National Yang Ming Chiao Tung University, Taipei 112, Taiwan; 6 Ph.D. Program for Neural Regenerative Medicine, College of Medical Science and Technology, Taipei Medical University, Taipei 110, Taiwan; 7 Graduate Institute of Medical Sciences, College of Medicine, Taipei Medical University, Taipei 110, Taiwan; 8 Division of Pulmonary Medicine, Department of Internal Medicine, School of Medicine, College of Medicine, Taipei Medical University, Taipei 110, Taiwan; 9 Division of Pulmonary Medicine, Department of Internal Medicine, Shuang Ho Hospital, Taipei Medical University, New Taipei City 235, Taiwan; 10 Division of Pulmonary Medicine, Department of Internal Medicine, Wan Fang Hospital, Taipei Medical University, Taipei 110, Taiwan; 11 Cell Physiology and Molecular Image Research Center, Wan Fang Hospital, Taipei Medical University, Taipei 110, Taiwan

**Keywords:** COPD, emphysema, hippo pathway, inflammation, lungs

## Abstract

Chronic obstructive pulmonary disease (COPD) is a major cause of morbidity and mortality in chronic lung disease patients throughout the world. Mesenchymal stem cells (MSCs) have been shown to regulate immunomodulatory, anti-inflammatory, and regenerative responses. However, the effects of human-umbilical-cord-derived mesenchymal stem cells (hUC-MSCs) on the lung pathophysiology of COPD remain unclear. We aimed to investigate the role of hUC-MSCs in emphysema severity and Yes-associated protein (Yap) phosphorylation (p-Yap) in a porcine-pancreatic-elastase (PPE)-induced emphysema model. We observed that the emphysema percentages (normalized to the total lung volume) measured by chest computed tomography (CT) and exercise oxygen desaturation were significantly reduced by hUC-MSCs at 10^7^ cells/kg body weight (BW) via intravenous administration in emphysematous mice (*p* < 0.05). Consistently, the emphysema index, as assessed by the mean linear intercept (MLI), significantly decreased with hUC-MSC administration at 3 × 10^6^ and 10^7^ cells/kg BW (*p* < 0.05). Changes in the lymphocytes, monocytes, and splenic cluster of differentiation 4-positive (CD4^+^) lymphocytes by PPE were significantly reversed by hUC-MSC administration in emphysematous mice (*p* < 0.05). An increasing neutrophil/lymphocyte ratio was reduced by hUC-MSCs at 3 × 10^6^ and 10^7^ cells/kg BW (*p* < 0.05). The higher levels of tumor necrosis factor (TNF)-α, keratinocyte chemoattractant (KC), and lactate dehydrogenase (LDH) in bronchoalveolar lavage fluid (BALF) were significantly decreased by hUC-MSC administration (*p* < 0.05). A decreasing p-Yap/Yap ratio in type II alveolar epithelial cells (AECII) of mice with PPE-induced emphysema was significantly increased by hUC-MSCs (*p* < 0.05). In conclusion, the administration of hUC-MSCs improved multiple pathophysiological features of mice with PPE-induced emphysema. The effectiveness of the treatment of pulmonary emphysema with hUC-MSCs provides an essential and significant foundation for future clinical studies of MSCs in COPD patients.

## 1. Introduction

Chronic obstructive pulmonary disease (COPD) is a chronic lung disease characterized by a progressive and irreversible condition that is one of the leading causes of mortality worldwide [1,2]. Approximately 6% of all deaths (more than 3 million people) occurred as a result of COPD in 2012 [3]. COPD incidence is predicted to rise in the coming decades as a result of continued exposure to various risk factors and aging populations [4]. COPD patients with emphysema have a greater mortality rate, a higher frequency of exacerbations, and a lower quality of life [5]. Emphysema in COPD is caused by a chronic inflammatory response following the inhalation of cigarette smoke (CS) or other noxious external particles, such as air pollution and biomass fuel [3]. This inflammation is also characterized by increased numbers of alveolar macrophages, neutrophils, T lymphocytes, and innate lymphoid cells recruited from the circulation [6]. Despite inflammation being a fundamental aspect of the disease pathogenesis, most COPD patients do not respond well to corticosteroids and exhibit no lung structural improvement [7]. As there are still not many therapeutic options that can improve the disease, understanding the lung-repair mechanism and finding additional therapeutic management options for COPD are still required.

Mesenchymal stem cells (MSCs) are adult stem cells derived from a range of sources, including umbilical cord (UC) tissue, bone marrow, adipose tissues, and others, and they have recently been used for disease treatment [8,9,10]. For clinical applications, bone marrow or adipose tissue derived MSCs, for example, have drawbacks such as an invasive harvesting procedure, decreased proliferation, and a differentiation potential related to donor age and comorbidities [11]. UC-MSCs, on the other hand, which are isolated from umbilical cord connective tissue, are easily obtained and expanded in vitro, with a better proliferative capacity and cell viability than other MSCs [12,13]. Therefore, UC-MSC is an ideal source of MSCs for stem cell transplantation. MSCs can release a wide range of cell signaling cytokines and growth factors that target endogenous stem cell self-renewal and migration, and stimulate host stem cells to self-renew and differentiate following an injury [10]. In our previous study on mice with CS-induced emphysema, we showed that the administration of human (h)UC-MSCs decreased pulmonary inflammatory responses and the severity of CS-induced emphysema [14]. In addition, hUC-MSCs can also reduce the pulmonary inflammatory response and display antiapoptotic effects in C57BL/6 mice with pulmonary inflammation induced by acute CS exposure [15]. However, reports on systemic immune cell profiles in porcine pancreatic elastase (PPE)-induced emphysema and after hUC-MSC administration are still limited [16].

Lung regenerative processes involve local stem or progenitor cell populations such as type II alveolar epithelial cells (AECII) [17,18]. The functions of the Hippo signaling pathway in AECII were recently studied [19]. The Yes-associated protein (Yap) and transcriptional coactivator with the PDZ-binding motif (Taz) are transcriptional coactivators and are the main downstream mediators of the Hippo pathway [20]. When the Hippo pathway is inhibited, Yap/Taz accumulate in the nuclei and interact with transcription factors, activating the gene expressions associated with cell survival, proliferation, and differentiation [21]. A recent study also showed that Yap/Taz regulate AECII activities, including their proliferation and differentiation into type I alveolar epithelial cells (AECI), as well as inflammatory responses during homeostasis following lung injury [19]. This suggests that Yap/Taz may play an important role in regulating lung regeneration during lung diseases, including emphysema in COPD. However, the role of Yap regulation on AECII by hUC-MSCs in emphysema remains unclear. The aim of this study was to investigate the effect of hUC-MSC administration on the severity of emphysema, the immune cell profile, and Yap phosphorylation (p-Yap) in a PPE-induced emphysema model.

## 2. Results

### 2.1. Reduction of Emphysema Severity and Exercise Oxygen Desaturation by hUC-MSCs

**Figure 1:**
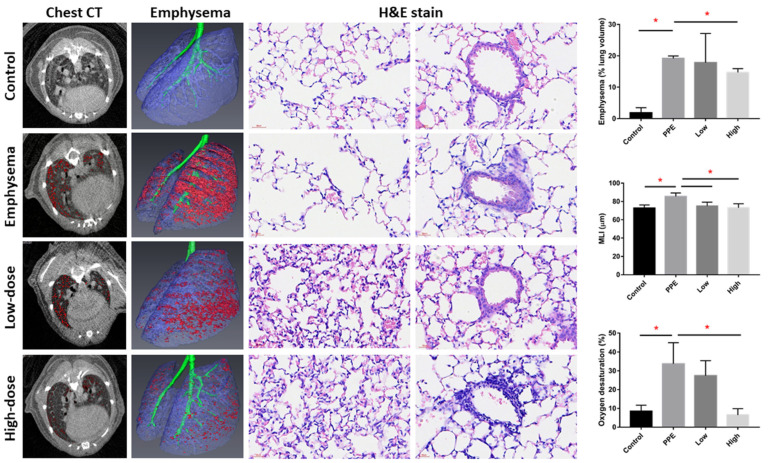
Human-umbilical-cord-derived mesenchymal stem cells (hUC-MSCs) improved emphysema severity and exercise oxygen desaturation in mice with porcine-pancreatic-elastase (PPE)-induced emphysema. Representative images of mouse chest micro-computed tomographic (CT) scans, reconstruction of lung emphysema, and alveolar and bronchial regions with H&E staining. The bars represent the quantification of emphysema percentages (normalized to the total lung volume), mean linear intercept (MLI), and oxygen desaturation after exercise. Administration of 3 × 10^6^ cells/kg body weight (low) and 10^7^ cells/kg body weight (high) hUC-MSCs decreased the emphysema percentage, MLI, and oxygen desaturation after exercise. * *p* < 0.05.

Figure 1 shows the emphysema severity and oxygen desaturation in mice with PPE-induced emphysema after hUC-MSC administration. Micro-CT scanning of the lung showed an increased emphysematous region (represented in red) in PPE-mice model compared with the control. The emphysema percentages (normalized to the total lung volume) significantly increased after PPE administration (*p* < 0.05). The emphysema percentages were significantly reduced by hUC-MSCs at a high concentration (14.7% ± 1.2%) in emphysematous mice (*p* < 0.05). In the hematoxylin and eosin (H&E) staining, increased alveolar space with a loss of alveolar attachments can be seen in the PPE-induced emphysema group compared with the control and hUC-MSCs administrated groups. Consistently, MLI was significantly increased by PPE (*p* < 0.05), which was significantly decreased by both low and high concentrations of hUC-MSC administration (*p* < 0.05). Additionally, oxygen desaturation after exercise increased by PPE compared with the control (*p* < 0.05). Increased oxygen desaturation levels were significantly reduced by the high concentration of hUC-MSCs (*p* < 0.05).

### 2.2. Lymphocyte Activation in the Spleen by hUC-MSCs

**Figure 2:**
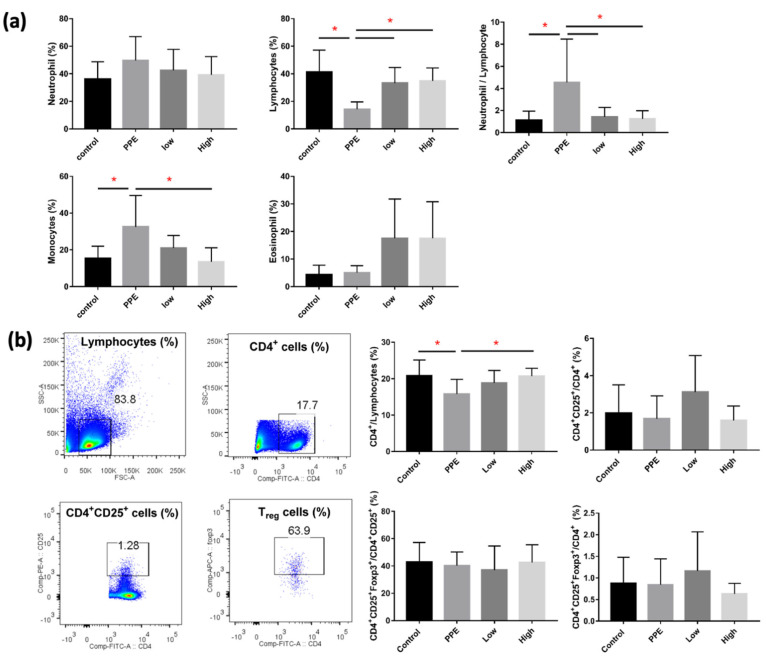
Human-umbilical-cord-derived mesenchymal stem cells (hUC-MSCs) reversed the inflammatory cell changes in mice with porcine-pancreatic-elastase (PPE)-induced emphysema. (**a**) Differential inflammatory cell counts in the bronchoalveolar lavage fluid (BALF). The administration of 3 × 10^6^ cells/kg body weight (low) and 10^7^ cells/kg body weight (high) hUC-MSCs reversed the lymphocyte, monocyte, and neutrophil/lymphocyte counts in BALF. (**b**) Quantification of lymphocyte alterations in the spleens of mice. Cluster of differentiation 4-positive (CD4^+^) cells increased after the administration of a high dose of hUC-MSCs. * *p* < 0.05.

We observed a significant decrease in lymphocytes and a significant increase in monocytes in mice with PPE-induced emphysema (*p* < 0.05) (Figure 2a). The decrease in lymphocytes and increase in monocytes were significantly reversed by hUC-MSC administration (both low and high concentrations for lymphocytes and the high concentration for monocytes) (*p* < 0.05). NLR significantly increased after PPE administration, which was reduced by hUC-MSCs at both low and high concentrations (*p* < 0.05). Figure 2b shows alterations in the lymphocyte in the spleen by hUC-MSCs in mice with PPE-induced emphysema. We observed that CD4^+^ lymphocytes were significantly decreased by PPE; however, the decreasing level was significantly increased by a high concentration of hUC-MSCs (*p* < 0.05). We did not observe a significant change in CD4^+^ CD25^+^ cells or regulatory T (Treg) cells in the spleen.

### 2.3. Reduction of Inflammation by hUC-MSCs

**Figure 3:**
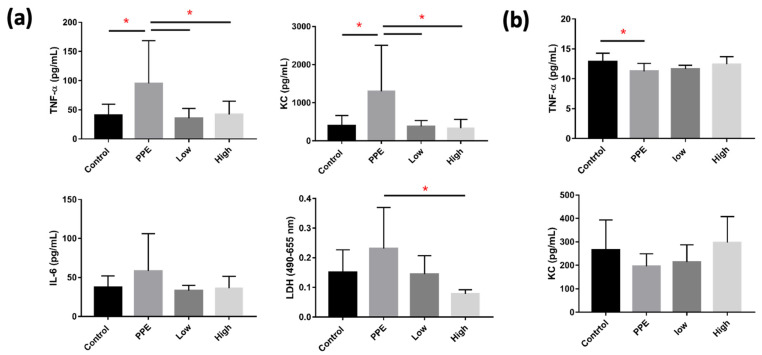
Human-umbilical-cord-derived mesenchymal stem cells (hUC-MSCs) decreased inflammatory cytokines in mice with porcine-pancreatic-elastase (PPE)-induced emphysema. (**a**) Administration of 3 × 10^6^ cells/kg body weight (low) and 10^7^ cells/kg body weight (high) hUC-MSCs reduced tumor necrosis factor (TNF)-α, keratinocyte chemoattractant (KC), and lactic dehydrogenase (LDH) inflammatory cytokine concentrations in the bronchoalveolar lavage fluid (BALF). (**b**) TNF-α was reduced in the serum of mice with PPE-induced emphysema. * *p* < 0.05.

Levels of TNF-α, KC, and LDH in BALF significantly increased with PPE, but decreased with hUC-MSC administration (*p* < 0.05) (Figure 3a). However, we did not observe a significant change in IL-6 in BALF between the groups. TNF-α in the serum was significantly decreased by PPE (*p* < 0.05), but this was not significantly reversed by hUC-MSC administration (Figure 3b). The KC, however, was not significantly different between the groups.

### 2.4. Increase in p-Yap of AECII by hUC-MSCs

**Figure 4:**
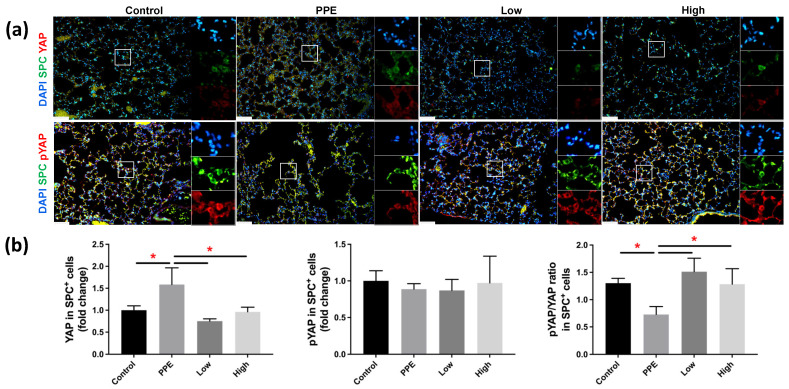
Human-umbilical-cord-derived mesenchymal stem cells (hUC-MSCs) increased yes-associated protein (Yap) phosphorylation in mice with porcine-pancreatic-elastase (PPE)-induced emphysema. (**a**) Representative immunofluorescence staining images of the lung alveolar regions. (**b**) Bars represent the quantification of Yap and phosphorylated (p)-Yap mean fluorescence intensity in alveolar epithelial type II (AECII) specific surfactant protein C (SPC^+^) cells. The administration of 3 × 10^6^ cells/kg body weight (low) and 10^7^ cells/kg body weight (high) hUC-MSCs decreased the Yap expression and increased the p-Yap/Yap expression in AECII SPC^+^ cells. Scale bar: 50 μm. * *p* < 0.05.

Figure 4a shows the Yap and p-Yap expressions in SPC^+^ AECII cells in mice with PPE-induced emphysema after hUC-MSC administration. We observed a significant increase in Yap expression in the SPC^+^ cells of mice with PPE-induced emphysema, which was reduced following hUC-MSC administration at both low and high concentrations (*p* < 0.05) (Figure 4b). The ratio of p-Yap to Yap expressions in SPC^+^ cells as significantly decreased in mice with PPE-induced emphysema, and increased upon the administration of both low and high concentrations of hUC-MSC (*p* < 0.05).

## 3. Discussion

The novelty of this study is that we investigated the effects of hUC-MSC administration on emphysema severity and the lung molecular pathophysiology in a PPE-induced emphysema model. The significant findings of our study were: (1) hUC-MSCs decreased the emphysema severity, (2) hUC-MSCs reduced exercise oxygen desaturation and inflammation, and (3) hUC-MSCs increased Yap phosphorylation on AECII cells in the PPE-induced emphysema mice model.

We observed significantly increased emphysema severity (CT scans and MLI) and exercise oxygen desaturation in mice with PPE-induced emphysema. Another study showed that PPE caused increased emphysema severity, as assessed by the MLI in mice [22]. A reduction in the lung parenchymal density, as determined by magnetic resonance imaging (MRI), was also previously reported in mice with PPE-induced emphysema [23]. This can be explained by the loss of alveolar walls and partial destruction of the capillary bed. In our study, emphysema was also evident in our functional assessment, in which significant oxygen desaturation was observed after exercise. This suggests an imbalance between the oxygen supply and demand during exercise, which can be attributed to inefficient gas exchange due to emphysema [24]. We further observed that the PPE-induced deterioration in lung function and structure improved after hUC-MSC administration. The recovery was reflected by significant decreases in emphysema severity and exercise oxygen desaturation. Our previous study using hUC-MSCs in a CS-induced mouse model also showed an improvement of emphysema upon hUC-MSC administration [14]. Another study using bone marrow-derived (BM)-MSCs showed a decrease in emphysematous lesions in the lungs of mice induced by PPE by intranasal instillation [25]. MSC administration can lead to the secretion of growth factors that have paracrine effects, which contribute to restoring the tissue architecture in the lungs [26]. While those studies evaluated different MSC sources and their effects on emphysema, the mechanisms of hUC-MSC interventions remain unclear.

Emphysema occurs as a result of persistent, low-level inflammation [27]. We observed alterations of the lung immune cell composition in PPE-induced emphysema, with increased monocytes and the NLR. COPD patients were also previously reported to have higher NLRs than healthy people, and those with a higher ratio had higher risks of exacerbation and need for hospitalization [28]. Neutrophil recruitment into the lung tissues contributes to disease development and progression, because these cells release several proteases, including neutrophil elastase, which lead to elastolysis and airspace enlargement [29]. Furthermore, elastase in the lungs can act as a chemoattractant for monocytes, attracting circulating monocytes into the lungs [30]. These monocytes undergo differentiation into pathogenic interstitial macrophages, which cause destruction of the alveolar walls and the development of emphysema. Together, this may explain the resulting emphysematous lungs caused by PPE in our study. In the present study, the administration of hUC-MSCs significantly reversed the immune cell profile in the lungs of PPE-induced emphysema, increasing the lymphocytes while decreasing the monocytes and NLR. MSCs were also previously reported to reduce the infiltration of neutrophils and macrophages into the lungs of an emphysema mouse model [22,31]. This therapeutic effect was thought to be due to the release of biological factors such as growth factors, cytokines, and extracellular vesicles, which promote wound healing [32]. MSC factors may also have a direct effect on intermediate cells such as monocytes, resulting in a reparative mechanism. Hence, this shows that administering hUC-MSCs can reduce pathogenic inflammatory cells in the lungs, which is consistent with our findings regarding improvements in emphysema and exercise oxygen desaturation.

We observed a decrease in CD4^+^ lymphocytes in the spleen of mice with PPE-induced emphysema. COPD patients were also reported to have lower proportions of circulating CD4^+^ T cells [33]. This decrease could be attributed to lymphocyte mobility from lymphoid organs to lung tissues, which is essential for stimulating lymphocyte differentiation and migration to target organs [22]. Furthermore, elastase in the lungs can interact with elastin receptors on recruited T cells, resulting in an inflammatory process related to emphysema [34]. As a result, the decrease in systemic CD4^+^ lymphocytes observed in our study may be associated with the onset of lung emphysema. We also discovered that hUC-MSC administration changed the immune cell profile, increasing the number of CD4^+^ lymphocytes in the spleen. A previous study of MSC administration in patients with severe emphysema also reported an increase in CD4^+^ lymphocytes [35]. However, there has been little study into the mechanism of MSC administration on CD4^+^ T cells in the pathogenesis of emphysema. Previous studies have shown that excessive lymphocyte migration from lymphoid organs was able to aggravate local inflammation and disease progression in the target organ [22,36]. MSC administration, on the other hand, can cause immunosuppression in lymphoid organs by arresting immature T cells in the G_0_/G_1_ phase of the cell cycle [37]. This reflects the findings in our study, in which the increase in spleen CD4^+^ lymphocytes upon hUC-MSC administration was associated with an improvement in emphysema.

We observed that mice with PPE-induced emphysema showed increased levels of TNF-α, KC, and LDH in BALF. Consistently, previous studies on emphysematous mouse models also revealed increased TNF-α and KC levels in the lungs [38,39,40]. TNF-α is a general proinflammatory marker, and KC is a known potent neutrophil chemoattractant [41]. These chemokine factors are produced as a result of elastolytic enzyme activity in lung epithelial cells and surface macrophages, and can draw circulating inflammatory cells into the lungs [27]. This may explain the presence of inflammatory cells and emphysema in the lungs of the mice in our study. We also found that TNF-α, KC, and LDH levels in BALF were also reduced by hUC-MSC administration. Similarly, decreases in TNF-α and KC levels were also reported in the lungs of emphysema mouse models upon the administration of hUC-MSCs [14,22]. MSCs decrease proinflammatory chemokines in the lungs, including TNF-α, KC, IL-1β, and matrix metalloproteinases (MMP)-12, which is attributed to increased secretion of transforming growth factor (TGF)-β [14,41,42]. This MSC-induced TGF-β secretion also reduces MMP-9 and MMP-12 expressions, which are responsible for alveolar destruction. Altogether, the effect of hUC-MSCs in reducing levels of proinflammatory chemokines in the lungs and inflammatory cell activation could explain the mitigation of emphysema in our study. The molecular pathway responsible for the repair of lung cells and structure, however, remains unclear.

Next, we observed decreased phosphorylation of Yap in AECII of this PPE-induced emphysema mouse model. Similarly, our previous study on an emphysema mouse model by air pollution also showed decreased Yap phosphorylation [43]. A previous study on mice with intranasal pneumococcal infection also found that infection increased AECII Yap nuclear activity [19]. Following lung injury, Yap phosphorylation was reduced, and it was translocated to the nuclei, where it regulated AECII functions such as proliferation and inflammatory responses [19]. AECII play important roles in alveolar homeostasis and regeneration after injury, as well as being a progenitor for AECI, and Yap is involved in this regulation [18,19]. Yap is a transcriptional coactivator that has been found to be distributed in both the nuclei and cytoplasm, and its activity primarily depends on phosphorylation-dependent Yap dynamic shifting [44]. When the Yap protein is not phosphorylated, it can shift to the nuclei and activate several mechanisms, including the promotion of proinflammatory responses [45,46]. Hence, we also evaluated the potential role of hUC-MSCs and observed that Yap phosphorylation subsequently increased in AECII upon hUC-MSC administration in mice with PPE-induced emphysema. A previous study in a mouse model of myocardial infarction revealed that Yap/Taz are required for an early inflammatory response mediated by macrophages, and that nuclear Yap upregulation increased proinflammatory responses and impaired reparative responses [46]. Those authors reported that the removal of Yap/Taz led to an anti-inflammatory phase dominated by reparative macrophages, which play an active role in tissue repair by promoting anti-inflammatory activities. This suggests that restoring the phosphorylation of Yap, or minimizing its transition to the nuclei, is essential for maintaining homeostasis and can attenuate early inflammatory responses. Our findings suggest that hUC-MSCs are involved AECII Yap phosphorylation to ameliorate PPE-induced emphysema.

This study has some limitations that should be considered. First, Yap expression should be confirmed with a Western blot analysis. Because the pathogenesis of emphysema is unclear, more cellular and molecular studies are needed to evaluate the changes in inflammatory cells and the signaling pathways involved. Additionally, only a few specific cytokines and growth factors were evaluated; a broader range of mediators should be analyzed to provide a more complete understanding of the mechanisms associated with each cell type.

## 4. Materials and Methods

### 4.1. Animals

Male 7-week-old C57BL/6JNarl mice obtained from the National Laboratory Animal Center (Taipei, Taiwan) were housed in the laboratory animal center of Taipei Medical University under 22 ± 2 °C, 55% ± 10% humidity, and 12 h dark/light cycle conditions. This study was performed under the approval of the Institutional Animal Care and Use Committee of Taipei Medical University (IACUC No. LAC-2019-0137).

### 4.2. Emphysema Mouse Model

**Figure 5:**
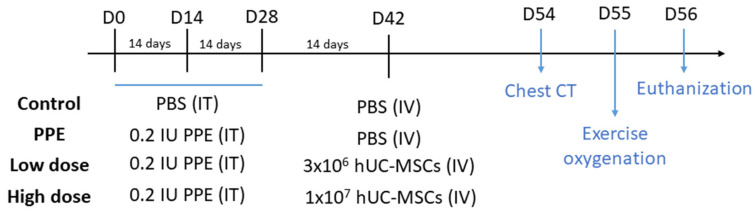
Schematic mouse model of the experimental design of porcine-pancreatic-elastase (PPE)-induced emphysema. The mice were intratracheally (IT) instilled three times with either phosphate-buffered saline (PBS) or 0.2 IU PPE (days 0, 14, and 28). Intravenous (IV) PBS or human umbilical cord-derived mesenchymal stem cells (hUC-MSCs) were administered 2 weeks later. Chest computed tomography (CT) and exercise oxygen saturation were assessed on days 54 and 55, respectively, and then the mice were sacrificed on day 56.

All of the mice were randomized by body weight (BW) before the experiment. The emphysema model was built by intratracheal (IT) instillation of 0.2 IU PPE (Sigma-Aldrich, St. Louis, MO, USA) using a microsprayer aerosolizer (Penn-Century, Wyndmoor, PA, USA) three times at a 2-week interval (on days 0, 14, and 28) under general anesthesia with 3% isoflurane using a rodent anesthesia machine (Northern Vaporisers; Skipton, UK) (Figure 5). The mice in the control group were given the same amount of phosphate-buffered saline (PBS) by IT instillation three times with the same intervals.

### 4.3. hUC-MSC Preparation

hUC-MSCs (Meridigen Biotech, Taipei, Taiwan) were harvested from UC tissues and cultured in a α-minimal essential culture medium (Invitrogen, Waltham, MA, USA) containing 18% fetal bovine serum (FBS; Invitrogen), 4 ng/mL basic fibroblast growth factor (Peprotech, Rocky Hill, NJ, USA), and 50 mg/mL gentamicin. The cells were incubated in a humidified air incubator at 37 °C (in 5% CO_2_ and 95% humidity). Six generations of cells were used throughout the study. Details of the hUC-MSC preparation and characterization were described previously [14,15]. Sample collection with written informed consent was carried out in accordance with the study protocol approved (A-BR-104-045) by the Ethics Committee of the National Cheng Kung University Hospital Institutional Review Board.

### 4.4. Experimental Design

Figure 5 shows the schematic experimental design of this study. After PPE or PBS treatment (on day 42), the mice were randomly assigned to four different groups: a control group (*n* = 10), a PPE group (*n* = 10), a low-dose hUC-MSC group (Low) (*n* = 10), and a high-dose hUC-MSC group (High) (*n* = 10). The mice in the hUC-MSC groups were administered hUC-MSCs at a dose of 3 × 10^6^ cells/kg BW for low-dose group and 10^7^ cells/kg BW for the high-dose group via tail vein administration. The hUC-MSC dosages were referenced from our previous report [14]. The mice in the control and emphysema groups were administered 300 μL of vehicle. At 2 weeks after hUC-MSC administration (days 54 and 55), chest computed tomography (CT) and exercise oxygenation were conducted. On day 56, the mice were euthanized, and the bronchoalveolar lavage fluid (BALF), serum, lungs, and spleen were collected. Details of sample preparation were previously reported [15,47].

### 4.5. Micro-CT

The mice were anesthetized and placed in a chamber of an in vivo micro-CT instrument (Skyscan 1176, Kontich, Belgium). The micro-CT scanner was periodically calibrated according to the manufacturer’s instructions. A water-containing Eppendorf tube was used as a phantom to calibrate the image correlated with Hounsfield units (HU). Images were acquired in list mode with the following parameters: an X-ray source voltage of 50 kVp, a current of 500 μA, a composite X-ray filter of 0.5 mm aluminum, a camera exposure time of 87 ms per projection, projections acquired with 0.7° increments over a total angle of 180°, and images produced with a real pixel size of 34.75 µm. The reconstructed images had a total of 573 slices with an isotropic 34.79 μm voxel size and an image resolution of 864 × 852.

### 4.6. Micro-CT Image Quantitative Analysis

Avizo 7.0, for 3D visualization and analysis (FEI, Visualization Sciences Group, Burlington, MA, USA), was adopted to perform a quantitative analysis of the extent of emphysema on the CT images. First, we used a threshold procedure to extract the entire lung field from micro-CT images. The selected threshold was set in the range of −900 to −200 HU, because intensity values below −900 HU are rare in the scans of healthy mice (the volume below −900 HU was <5% of the total lung volume in all healthy animals of any age) [48]. Then, the airways were segmented using a region-growing method for airway tree segmentation [49]. The algorithm is based on an initial candidate region of an airway from micro-CT images by planting a seed and propagating a voxel comparison algorithm that automatically searches the second candidate region. As the seed grows, it connects to similar voxels of adjacent regions and obtains a three-dimension model of the airway branches. The airway branches were removed from the whole lung volume before further emphysema quantification, because previous reports revealed that areas of lower than −600 HU significantly increased in parallel with the degree of emphysematous characteristics of the lungs [50]. The final step was to set the low attenuation area (LAA) from −871 to −610 HU as the area of emphysema, and to produce segments for three-dimensional (3D) quantification. The obtained area of the emphysema was normalized to the total lung volume and was displayed in percentage for the analysis.

### 4.7. Exercise Oxygen Desaturation

Oxygen saturation was measured via the tail using a pulse oximetry system (BIOPAC System, Santa Barbara, CA, USA) for 3 min. Next, the mice were placed on a treadmill at a steady speed of 150 cm/min for 2 min of training and an additional 5 min of running. The difference in mean values between pre- and post-exercise was calculated to determine oxygen desaturation (%).

### 4.8. BALF Cell Counting

BALF cell counting was conducted using a hematology analysis (ProCyte Dx; IDEXX Laboratories; Westbrook, ME, USA). Neutrophils, monocytes, lymphocytes, and eosinophils were determined and presented as percentages of the total cell counts. The neutrophil to lymphocyte ratio (NLR) was calculated as an index for subclinical inflammation [51].

### 4.9. Flow Cytometry

The mouse splenocytes were isolated using an ammonium-chloride-potassium (ACK) buffer. To measure the expressions of cluster of differentiation 4 (CD4) and CD25 in the splenocytes, 10^6^ cells were incubated for 30 min at 4 °C with specific mouse antibodies (CD4 and CD25) conjugated with fluorochromes (fluorescein isothiocyanate (FITC), phycoerythrin (PE), or allophycocyanine (APC)). For intracellular staining, the cells were permeabilized with a Foxp3/Transcription factor staining buffer set (eBioscience, San Diego, CA, USA) and stained with a Foxp3 mouse antibody. Data from 10,000 events in live cells were analyzed with FlowJo software (TreeStar, Ashland, OR, USA). To understand the influence of the percentage of Foxp3-positive cells in CD4^+^CD25^+^ splenocytes, Foxp3^+^ cells were gated and transformed into a percentage of CD4^+^CD25^+^ splenocytes.

### 4.10. Biochemical Analysis

Interleukin (IL)-6 (ThermoFisher, Waltham, MA, USA), chemokine (CXC motif) ligand 1/keratinocyte chemoattractant (CXCL1/KC) (R&D Systems, Minneapolis, MN, USA), tumor necrosis factor (TNF)-α (ThermoFisher), and lactic dehydrogenase (LDH; ThermoFisher) in BALF and TNF-α and KC in serum were examined according to the manufacturers’ instructions.

### 4.11. Mean Linear Intercept (MLI)

Hematoxylin and eosin (H&E) staining of the lungs was performed according to our previous report [47]. MLI, an indicator of the mean alveolar diameter, was assessed in 10 non-overlapping fields according to a previous report [48]. Histological examinations were conducted under light microscopy.

### 4.12. Immunofluorescence (IF) Staining

IF staining was performed on 4 µm lung slide sections from paraffin blocks, as previously reported [52]. Briefly, sections were deparaffinized and submitted for antigen retrieval before permeabilization by 0.25% Triton X-100/1% bovine serum albumin (BSA)/PBS and blocking (30 min in 5% BSA/PBS). Primary antibodies SFTPC (#32459, Signalway, Greenbelt, MD, USA), Yap (66900-1-Ig, Proteintech, Rosemont, IL, USA), phosphorylated (p)-Yap (AB76252, Abcam, Cambridge, UK), and secondary antibodies FITC-conjugated species-specific (AB150077 and AB150116, Abcam, Cambridge, UK) were used for IF staining. The nuclei were counterstained with 4′,6-diamidino-2-phenylindole (DAPI) (AB104139, Abcam). Images were captured using confocal fluorescence microscopy (TCS SP5, Leica, Wetzlar, Germany) equipped with a camera and imaging software (SPOT Imaging, Sterling Heights, MI, USA). The mean fluorescence intensity (MFI) of the Yap and p-Yap expressions in the specific surfactant protein C-positive (SPC^+^) AECII in five different lung alveolar regions were analyzed. Image analysis was performed with ImageJ software, as described in a previous report [53].

### 4.13. Statistical Analysis

All data are expressed as the mean ± standard deviation (SD). Comparisons within multiple groups were performed using an analysis of variance (ANOVA) with Tukey’s post hoc test. Statistical analyses were performed using GraphPad vers. 8.2.1 for Windows 10. The significance criterion was set to *p* < 0.05.

## 5. Conclusions

In conclusion, hUC-MSCs reduced emphysema severity and inflammatory responses in mice with PPE-induced emphysema, which involved Yap phosphorylation in resolving emphysema. Our findings suggest that understanding the mechanism of the hUC-MSC intervention on immune cell responses and the Hippo signaling pathway could be a crucial contributor to achieving regeneration in alveolar destruction due to emphysema.

## Data Availability

The datasets used and/or analyzed during the current study are available from the corresponding author upon reasonable request.

## Abbreviations

AECI	Type 1 alveolar epithelial cells
AECII	Type 2 alveolar epithelial cells
BALF	Bronchoalveolar lavage
CD	Cluster of differentiation
COPD	Chronic obstructive pulmonary disease
CS	Cigarette smoke
CT	Computed tomography
H&E	Hematoxylin and eosin
hUC-MSC	Human umbilical cord mesenchymal stem cell
IT	Intratracheal
KC	Keratinocyte chemoattractant
LDH	Lactic dehydrogenase
MFI	Mean fluorescence intensity
MLI	Mean linear intercept
NLR	Neutrophil to lymphocyte ratio
PBS	Phosphate-buffered saline
PPE	Porcine-pancreatic elastase
SPC	Surfactant protein C
TAZ	transcriptional coactivator with PDZ-binding motif
TNF-α	Tumor necrosis factor α
YAP	Yes-associated protein
